# Induction of the Prenylated Stilbenoids Arachidin-1 and Arachidin-3 and Their Semi-Preparative Separation and Purification from Hairy Root Cultures of Peanut (*Arachis hypogaea* L.)

**DOI:** 10.3390/molecules27186118

**Published:** 2022-09-19

**Authors:** Amit Raj Sharma, Gaurav Gajurel, Izzeldin Ahmed, Krystian Roedel, Fabricio Medina-Bolivar

**Affiliations:** 1 Arkansas Biosciences Institute, Arkansas State University, Jonesboro, AR 72467, USA; 2 Department of Biological Sciences, Arkansas State University, Jonesboro, AR 72467, USA; 3 Molecular Biosciences Graduate Program, College of Sciences and Mathematics, Arkansas State University, Jonesboro, AR 72467, USA

**Keywords:** hairy root, peanut, prenylated stilbenoid, arachidin-1, arachidin-3, elicitation, re-elicitation, methyl jasmonate, cyclodextrin, hydrogen peroxide

## Abstract

Prenylated stilbenoids such as arachidin-1 and arachidin-3 are stilbene derivatives that exhibit multiple pharmacological activities. We report an elicitation strategy using different combinations of cyclodextrin, hydrogen peroxide, methyl jasmonate and magnesium chloride to increase arachidin-1 and arachidin-3 production in peanut hairy root cultures. The treatment of hairy root cultures with cyclodextrin with hydrogen peroxide selectively enhanced arachidin-1 yield (132.6 ± 20.4 mg/L), which was 1.8-fold higher than arachidin-3. Similarly, cyclodextrin combined with methyl jasmonate selectively enhanced arachidin-3 yield (178.2 ± 6.8 mg/L), which was 5.5-fold higher than arachidin-1. Re-elicitation of the hairy root cultures further increased the levels of arachidin-1 and arachidin-3 by 24% and 42%, respectively. The ethyl acetate extract of the culture medium was consecutively fractionated by normal- and reversed-phase column chromatography, followed by semi-preparative HPLC purification on a C18 column to yield arachidin-1 with a recovery rate of 32% and arachidin-3 with a recovery rate of 39%, both at higher than 95% purity. This study provided a sustainable strategy to produce high-purity arachidin-1 and arachidin-3 using hairy root cultures of peanuts combined with column chromatography and semi-preparative HPLC.

## 1. Introduction

**Figure 1:**
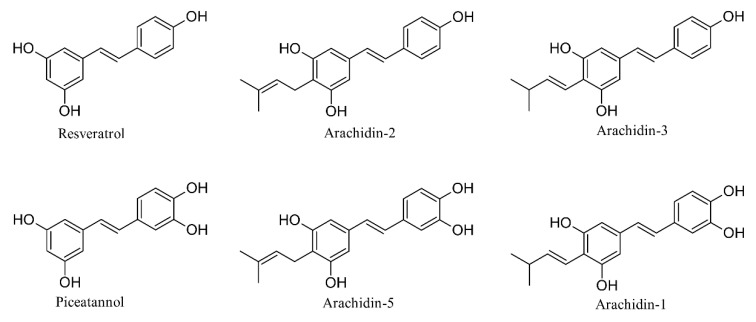
Chemical structures of resveratrol and piceatannol and their prenylated analogs.

Peanut (*Arachis hypogaea* L.) is known to produce stilbenoid-type phytoalexins upon exposure to biotic and abiotic stresses. The major phytoalexins include the non-prenylated stilbenoid resveratrol and the prenylated stilbenoids arachidin-1 and arachidin-3 [1,2,3] (Figure 1). Prenylation is the process of adding a prenyl (C5) moiety to the backbone structure of a chemical. In the peanut, it usually occurs at the C-4 position of the stilbene through the action of a stilbene-specific prenyltransferase [4]. Prenylation plays a major role in bioactivity by increasing the lipophilicity and affinity of natural products to the cell membrane [5]. Prenylated aromatic compounds have special therapeutic significance owing to their various pharmacological activities including anticancer and anti-inflammatory effects that are interesting for industrial applications, though their exploitation is limited due to their low abundance in nature [6,7].

Arachidin-1 and arachidin-3 are prenylated analogs of piceatannol and resveratrol, respectively. They exhibit a 3-methyl-1-butenyl moiety rather than the typical 3,3-dimethylallyl moiety as found in arachidin-2 and arachidin-5 (Figure 1). Both arachidin-1 and arachidin-3 have shown anti-inflammatory, antiviral, antibacterial, antioxidant, antiadipogenic, and antitumorigenic effects in in vitro studies [3,8,9,10,11,12,13,14]. Recently, arachidin-1 was shown to exhibit potent antiproliferative activity in triple-negative breast cancer cells at low micromolar concentrations and it was not toxic to non-cancerous breast cells at these concentrations [15]. In addition, arachidin-1 and arachidin-3 exhibited a higher binding affinity for cannabinoid receptors when compared to their non-prenylated parent compounds piceatannol and resveratrol. Furthermore, the prenyl unit in arachidin-1 and arachidin-3 prevented the formation of glucuronide derivatives in vitro, which indicates the potential for slower metabolism and enhanced bioavailability than their non-prenylated analogs [12]. Most of the biological activities of arachidin-1 and arachidin-3 were demonstrated in vitro. Studies in vivo are limited because these compounds are not commercially available. Therefore, this study aimed to develop a strategy to produce arachidin-1 and arachidin-3 at high levels and purity amenable for in vivo testing.

To overcome the supply problem for arachidin-1 and arachidin-3, different strategies for high-level production of stilbenoids are required. Our laboratory established peanut hairy root cultures as an elicitor-controlled bioproduction platform for prenylated stilbenoids [2,16,17,18]. Hairy root cultures are produced via *Agrobacterium rhizogenes*-mediated transformation. These tissue cultures are unique due to their ability to grow rapidly in a phytohormone-free medium and long-term biochemical stability. Elicitation is a strategy used to initiate or improve the biosynthesis of specialized metabolites in various plants by stimulating stress responses with the addition of elicitors. Thus, the combination of elicitors methyl jasmonate (MeJA), methyl-β-cyclodextrin (CD), and hydrogen peroxide (H_2_O_2_) with MgCl_2_ stimulated the production of prenylated stilbenoids in hairy root cultures of peanut [18].

The semi-preparative separation and isolation of prenylated stilbenoids are a significant challenge because of their low abundance in nature [7]. In the past, the isolation of peanut stilbenoids was based on chromatography in silica gel and preparative HPLC [19,20]. However, methods for producing large amounts of purified peanut prenylated stilbenoids have not been described. This is the first study to report the purification of large quantities of prenylated stilbenoids arachidin-1 and arachidin-3 from peanut hairy root cultures using conventional chromatographic techniques and semi-preparative HPLC.

In the present study, we selectively enhanced the production of arachidin-1 and arachidin-3 in peanut hairy root cultures by treatment with different elicitor combinations. We also report easy-to-adapt and efficient normal- and reversed-phase column chromatographic techniques followed by a reversed-phase semi-preparative HPLC approach for the separation and purification of two major prenylated peanut stilbenoids, i.e., arachidin-1 and arachidin-3, from elicited hairy root cultures.

## 2. Results

### 2.1. Growth Kinetics of Peanut Hairy Roots in 500 mL Flasks

**Figure 2:**
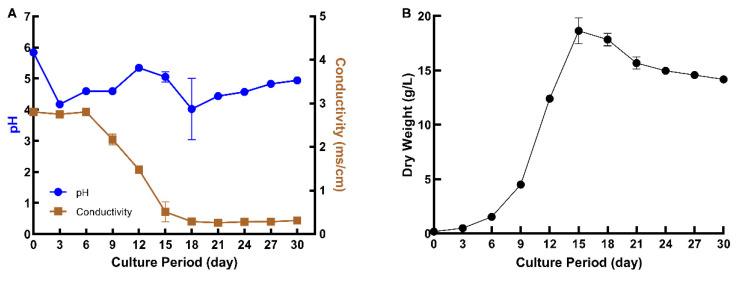
Growth kinetics of hairy roots of peanut. (**A**) Conductivity (brown) and pH of the medium (blue) at different stages of growth. (**B**) Growth curve of the hairy roots. Values represent the average of three biological replicates and the error bar represents the standard deviation.

Previously, hairy roots of peanut were cultured in 250 mL flasks with 50 mL of MSV medium [2,17,18]. However, preliminary experiments suggested that a higher biomass of roots could be obtained by culturing the same amount of root inoculum into larger flasks with a higher volume of the medium. Therefore, the hairy roots were cultured in 500 mL flasks with 100 mL of MSV medium, and a thirty-day growth curve was established to identify the growth stages of the hairy roots grown under these conditions. The pH and conductivity of the medium were recorded during the 30 days to understand the behavior of the roots in the medium (Figure 2A). The conductivity of the medium decreased as the biomass increased, indicating that nutrients were taken up from the culture medium by the hairy roots during their growth. The rate of change in conductivity leveled off after the hairy roots reached their highest biomass on day 15. The pH dropped for the first three days, slowly increased for the next nine days, and then decreased again on day 18. From day 18 to the end of the growth curve period, the pH slowly increased. Based on the dry weight of the hairy roots, a lag phase of about 3 days was observed, followed by an exponential growth phase between days 3–15 and a stationary phase from day 15 to 30 (Figure 2B). The specific growth rate, growth index, and doubling times were calculated as 0.31 day^−1^, 107.33, and 2.22 days, respectively. The highest biomass attained was 18.63 ± 1.19 g DW/L at 15 days. The specific growth rate was found to be similar to that described for peanut hairy roots grown in 250 mL flasks [17].

### 2.2. Phenotype of Hairy Root Cultures upon Multiple Elicitor Treatments

**Figure 3:**
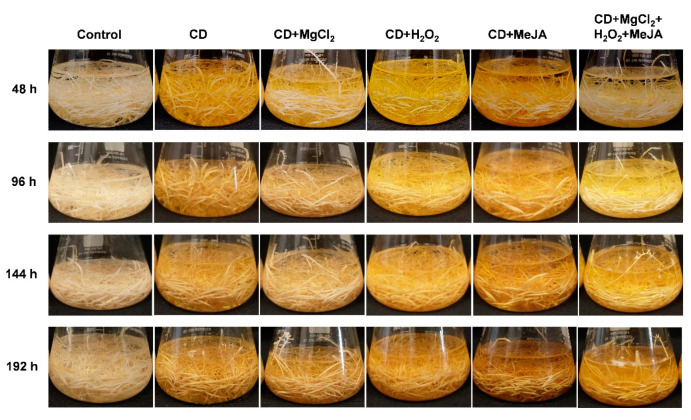
Phenotype of hairy root cultures of peanut upon treatment with different combinations of elicitors for 48, 96, 144, and 192 h. CD: methyl-β-cyclodextrin; MgCl_2_: magnesium chloride; H_2_O_2_: hydrogen peroxide; MeJA: methyl jasmonate.

To selectively enhance the production of the prenylated stilbenoids arachidin-1 and arachidin-3, 12-day-old peanut hairy root cultures were treated with a combination of elicitors for different time points (48, 96, 144, and 192 h). Interestingly, peanut hairy root cultures co-treated with CD+MeJA, and CD+MgCl_2_+H_2_O_2_+MeJA showed the most intense color after 192 h elicitation (Figure 3). The color of the culture medium turned from clear as observed in the non-treated control hairy root cultures to a dark yellow color upon the elicitation, which is indicative of the secretion of specialized metabolites into the medium. In addition, the hairy root tissue showed a brownish-yellow color after the elicitor treatment compared with the non-treated control group.

### 2.3. Effect of Different Elicitor Treatments on Yield of Archidin-1 and Arachidin-3

**Figure 4:**
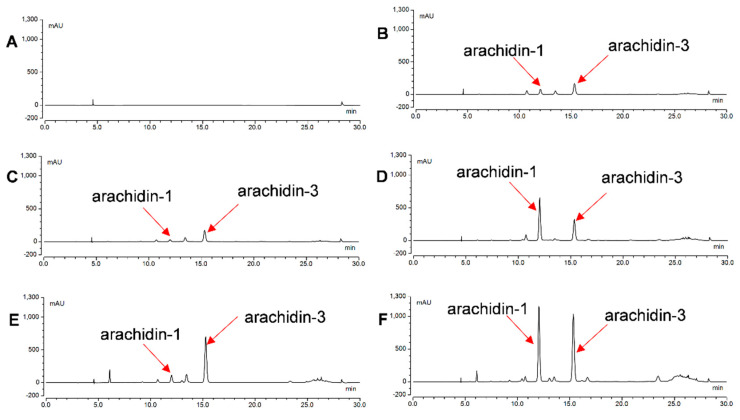
HPLC chromatograms of extracts from the medium of hairy root cultures of peanut upon elicitation treatment for 192 h with (**A**) control (without elicitors); (**B**) CD; (**C**) CD+MgCl_2;_ (**D**) CD+H_2_O_2;_ (**E**) CD+MeJA; and (**F**) CD+MgCl_2_+H_2_O_2_+MeJA. All chromatograms were monitored at 340 nm. CD: methyl-β-cyclodextrin; MeJA: methyl jasmonate.

**Figure 5:**
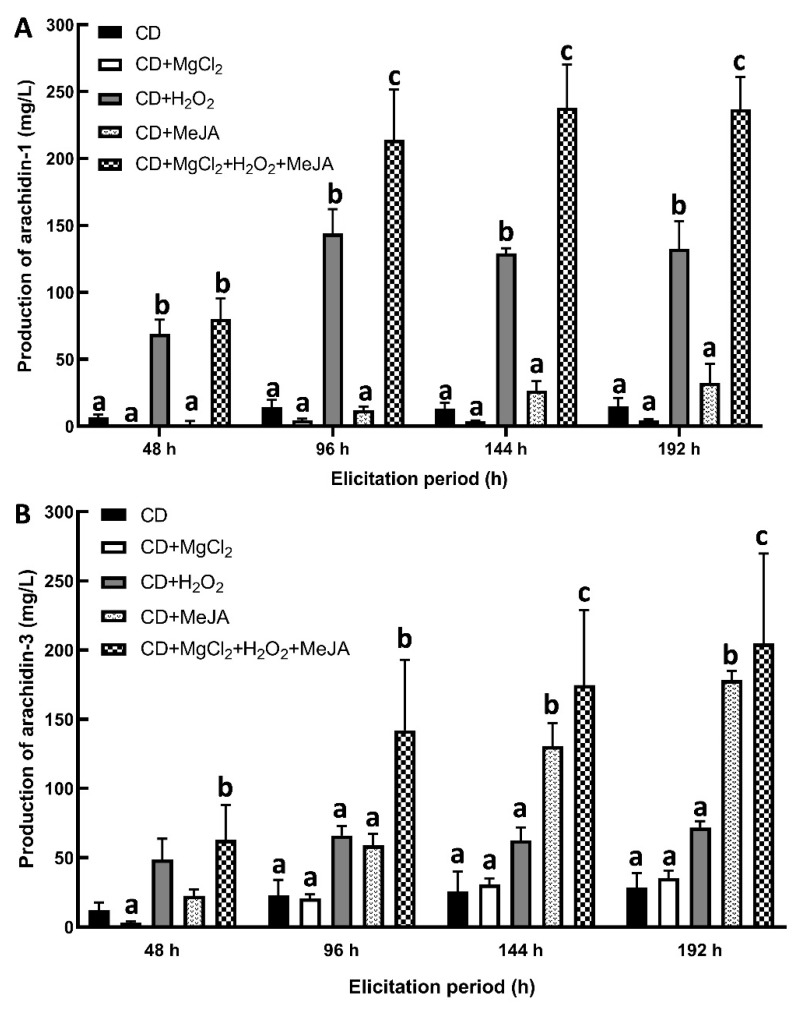
Comparison of (**A**) arachidin-1 and (**B**) arachidin-3 yield in hairy root cultures of peanut upon different elicitor treatments. Yield is expressed in mg/L of medium and each bar represents the average of three biological replicates and error bars represent standard deviation. Statistical analysis was performed using two-way ANOVA with Tukey’s multiple comparisons test. In each elicitation period, the lower-case letters above the column represent significant (between different letters) or non-significant (between the same letter) statistical differences. Significance level, *p* < 0.05. CD: methyl-β-cyclodextrin; MeJA: methyl jasmonate.

To determine the yield of arachidin-1 and arachidin-3 during the elicitation period, ethyl acetate extracts from the culture medium after 48, 96, 144, and 192 h of elicitor treatment were analyzed by HPLC (Figure 4A–F). Arachidin-1 and arachidin-3 were detected in the medium of all the elicited cultures. However, the levels differed significantly among particular elicitor treatments (Figure 5A,B). As described in previous studies with hairy root cultures of peanut, arachidin-1, and arachidin-3 were not detected in the non-treated control group [2]. Previous studies showed the production of prenylated stilbenoids arachidin-1 and arachidin-3 in the hairy root culture media of peanuts after the elicitation by different chemical elicitors [2,18]. These results showed that arachidin-1 and arachidin-3 are inducible specialized metabolites only secreted into the medium after the stress caused by elicitors.

The overall yield of arachidin-1 (236.8 ± 20 mg/L) and arachidin-3 (204.9 ± 64.8 mg/L) was higher in the peanut hairy root cultures medium co-treated with CD+MgCl_2_+H_2_O_2_+MeJA after a 192 h treatment (Figure 5A,B). The yield of arachidin-1 was approximately 1.1-fold higher than arachidin-3. Interestingly, the selective accumulation of arachidin-1 was found in the CD+H_2_O_2_ co-treated peanut hairy root cultures after 192 h. The production of arachidin-1 was 132.6 ± 20.4 mg/L, which was approximately 1.8-fold higher than arachidin-3. Similarly, the selective production of arachidin-3 was higher in the CD+MeJA co-treated peanut hairy root cultures after 192 h. The production of arachidin-3 was 178.2 ± 6.8 mg/L, which was approximately 5.5-fold higher than arachidin-1. The lowest amount of arachidin-1 (4.24 ± 1.1 mg/L) was detected in the hairy root culture medium co-elicited with CD+MgCl_2_, whereas the lowest amount of arachidin-3 (28.6 ± 10.4 mg/L) was found in hairy root culture elicited with CD only after 192 h. The selective increment in the production of targeted metabolites arachidin-1 (CD+H_2_O_2_ treatment) and arachidin-3 (CD+MeJA treatment) with low production of other stilbenoids can facilitate the semi-preparative separation and purification procedure for these prenylated stilbenoids.

### 2.4. Re-Elicitation of Elicited Peanut Hairy Root Cultures

**Figure 6:**
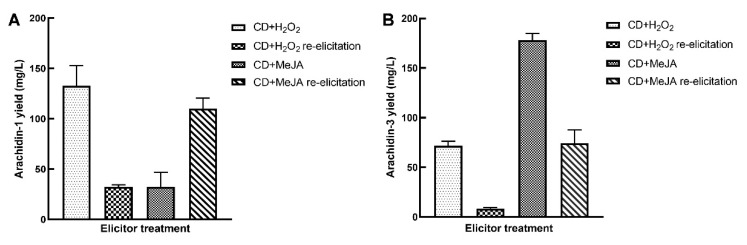
Comparison of (**A**) arachidin-1 and (**B**) arachidin-3 yield in hairy root cultures of peanut upon re-elicitation by CD+H_2_O_2_ and CD+MeJA. Yield is expressed in mg/L of medium and each bar represents the average of three biological replicates and error bars represent standard deviation. CD: methyl-β-cyclodextrin; MeJA: methyl jasmonate.

The capacity of the elicited hairy root cultures to respond to a second elicitor treatment was investigated by re-elicitation. After 192 h of elicitation, the hairy root cultures were re-elicited with the same elicitor combination, i.e., CD+H_2_O_2_ or CD+MeJA, for an additional 192 h. Re-elicited hairy root cultures co-elicited with CD+H_2_O_2_ showed a decreased amount of both arachidin-1 (32.3 ± 2.1 mg/L) and arachidin-3 (8.6 ± 0.8 mg/L) compared to the first elicitation (Figure 6A,B). Interestingly, re-elicited hairy root cultures co-elicited with CD+MeJA showed a decreased amount of arachidin-3 (74.5 ± 13.1 mg/L), whereas there was an enhanced production of arachidin-1 (110.3 ± 10.3 mg/L) compared to the first elicitation (Figure 6A,B). Overall, re-elicited hairy root cultures showed high production of arachidin-1 in both treatments compared to arachidin-3.

Our results showed that elicited hairy root cultures are amenable to elicitor response and can be used for re-elicitation which can reduce labor and cost and the overall time of stilbenoid production and purification process. To the best of our knowledge, this is the first report on the re-elicitation of peanut hairy root cultures with substantial production of arachidin-1 and arachidin-3. After the first 192 h elicitation period, the culture medium was replaced with a fresh medium or a medium with elicitors and the biomass was measured after an additional 192 h. Notably, the hairy root biomass was higher by 5.2% in the single-elicited hairy root cultures (replaced with fresh medium) when compared with the re-elicited hairy root cultures with CD+H_2_O_2_. Similarly, the biomass was higher by 3.2% in the single-elicited hairy root cultures (replaced with fresh medium) when compared with the re-elicited hairy root cultures with CD+MeJA. These results suggested that the hairy roots were still viable and could continue to grow after the first elicitor treatments.

### 2.5. Purification of Arachidin-1 and Arachidin-3

**Figure 7:**
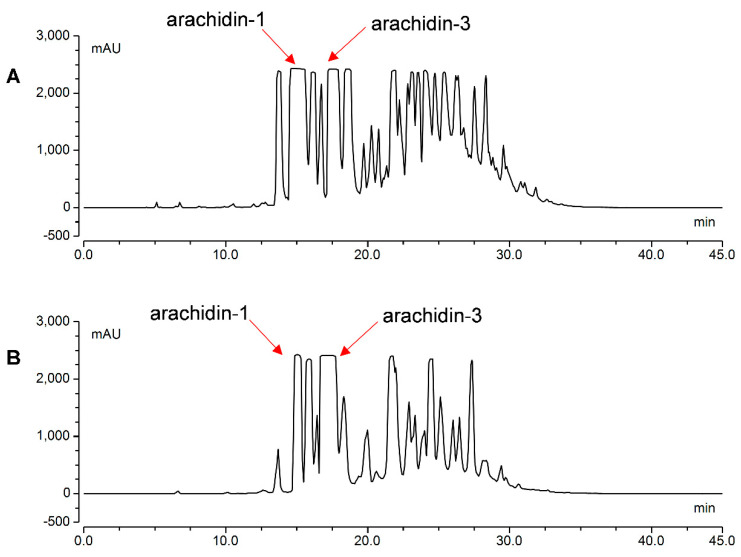
Semi-preparative HPLC chromatograms of 6:4 (MeCN:0.5%HCO_2_H) octadecylsilane (ODS) fraction from hairy root cultures of peanut elicited with (**A**) CD+H_2_O_2_ and (**B**) CD+MeJA. CD: methyl-β-cyclodextrin; MeJA: methyl jasmonate.

Column chromatography techniques followed by final purification by semi-preparative HPLC were performed to purify arachidin-1 and arachidin-3. To obtain the two major prenylated stilbenoids arachidin-1 and arachidin-3, peanut hairy root cultures were treated with CD+H_2_O_2_ or CD+MeJA. Our study showed that CD+H_2_O_2_ and CD+MeJA selectively enhanced the production of arachidin-1 and arachidin-3, respectively. However, overall production of arachidin-1 and arachidin-3 was higher in the all-elicitor (CD+MgCl_2_+H_2_O_2_+MeJA) treatment. In this study, we did not use the all-elicitor treated extract for further purification because it also included several other compounds that could potentially interfere with the purification process. The extract obtained from peanut hairy root cultures co-elicited with CD+H_2_O_2_ was used to obtain arachidin-1 (major target compound) along with arachidin-3. Similarly, the extract obtained from peanut hairy root cultures co-elicited with CD+MeJA was used to obtain arachidin-3 (major target compound) along with arachidin-1. The peanut hairy root cultures were elicited with CD+MeJA and CD+H_2_O_2_ for 192 h and the entire elicited culture medium was extracted with ethyl acetate. After evaporation, the extracts were consecutively fractionated by normal- and reversed-phase column chromatography techniques as described in the Section 4, followed by semi-preparative HPLC on a C18 column to yield arachidin-1 and arachidin-3 (Figure 7A,B). Arachidin-1 and arachidin-3 were obtained as an amorphous solid showing a white color with a yellow tint. Altogether, 295 mg of arachidin-1 and 530 mg of arachidin-3 were isolated by combining both elicitor treatments. To the best of our knowledge, this is the first report on the isolation and purification of arachidin-1 and arachidin-3 in large quantities. After the purification process, the purity of arachidin-1 and 3 was checked by HPLC-UV by the relative area covered by these compounds in the chromatogram. Arachidin-1 and arachidin-3 were obtained at over 95% purity (Supplementary Materials Figures S1 and S2).

### 2.6. Identification of Arachidin-1 and Arachidin-3

The identity of arachidin-1 and 3 was confirmed by comparison of HPLC retention time and UV spectrum with standards isolated and identified in our laboratory previously. Furthermore, mass spectrometry analysis of the purified compounds showed the same fragmentation pattern of arachidin-1 and arachidin-3 that was previously reported [21]. The mass spectrometry analysis of arachidin-1 showed a molecular ion at *m*/*z* 313 [M+H]^+^ and fragment ion at *m*/*z* 257 in MS^2^, suggesting the presence of a prenyl moiety. Similarly, mass spectrometry analysis of arachidin-3 showed a molecular ion at *m*/*z* 297 [M+H]^+^ and a fragment ion at *m*/*z* 241 in MS^2^, indicating the presence of a prenyl moiety (Table 1) (Supplementary Materials Figures S3–S6).

## 3. Discussion

Hairy root cultures were used as an attractive system for producing bioactive specialized metabolites owing to their characteristic high growth rate, genetic and biochemical stability, and similar or higher biosynthetic potential to parent plants [22]. Peanut hairy root cultures are currently attracting significant attention owing to their ability to produce bioactive prenylated stilbenoids. Indeed, the peanut hairy root cultures treated with various elicitors are known to produce the prenylated stilbenoids arachidin-1, arachidin-2, arachidin-3, and arachidin-5 [21]. However, most peanut prenylated stilbenoids were isolated from fungi-challenged peanut seeds. Thus, *Aspergillus* and *Rhizopus* species were used to challenge peanut seeds to produce novel stilbene phytoalexins and prenylated derivatives, including SB-1, chiricanine A, arahypin-1, arahypin-2, arahypin-3, arahypin-4, arahypin-5, arahypin-6 and arahypin-7 [3,20,23,24,25]. However, the drawbacks of producing stilbenoids from fungal-challenged peanut seeds are low yield, low recovery, modification of prenylated stilbenoids by fungi, and presence of mycotoxins [25].

Elicitation is a promising method to induce or enhance the production of secondary metabolites. In the present study, several combinations of elicitors including CD, MeJA, H_2_O_2_ and MgCl_2_ were tested to evaluate the capacity of peanut hairy root cultures to produce large quantities of prenylated stilbenoids arachidin-1 and arachidin-3. Cyclodextrins (CDs) are cyclic oligosaccharides derived from starch which are used as elicitors to increase the levels of secreted specialized metabolites in plant cells and tissue cultures by forming inclusion complexes with non-hydrophilic compounds. Furthermore, CDs are also commonly used to prevent feedback inhibition [26]. Methyl jasmonate (MeJA), a methyl ester of jasmonic acid, is an extensively used effective elicitor due to its outstanding role in signal transduction pathways. Indeed, various studies have demonstrated enhanced phytochemical contents in plants and hairy roots by MeJA treatment [27,28]. Hydrogen peroxide (H_2_O_2_) acts as a signal molecule that may trigger oxidative stress resulting in the production of specialized metabolites [29]. Magnesium (Mg^2^+) is the most abundant divalent cation in living cells and acts as a co-factor required for the activity of the prenyltransferases enzyme which plays a key role in the prenylation of [4,30].

Co-treatment with cyclodextrins and MeJA to hairy root cultures has emerged as a novel approach for the enhanced accumulation and induction of new secondary metabolites. Previously, taxol biosynthesis was increased by the joint action of cyclodextrins and MeJA by 55 times higher than in non-elicited *Taxus x media* cell cultures [31]. Increased production of alkaloids vindoline, catharanthine, and ajmalicine were reported in cell cultures of *Catharanthus roseus* co-treated with cyclodextrins and MeJA compared to individually treated cells [32]. This and previous studies showed the synergistic effect of cyclodextrins and MeJA in stilbenoid production in peanuts. However, the synergistic effect of CD+H_2_O_2_ has not been studied. The effect of exogenous H_2_O_2_ on the synthesis of stilbenoids was studied in *Vitis rotundifolia* hairy root cultures. H_2_O_2_ induced the secretion of resveratrol into the culture medium at higher levels than the other two major stilbenoids piceid and ε-viniferin [33]. In the present study, we treated peanut hairy root cultures with CD+H_2_O_2_ to investigate the induction of stilbenoid production in the culture medium, and interestingly, selectively enhanced production of arachidin-1 was found. On the other hand, CD+MeJA enhanced the production of arachidin-3. This difference in the accumulation of prenylated stilbenoids could be related to the distinct types of stress and signaling pathways induced by MeJA versus H_2_O_2_. For instance, the latter may increase oxidative stress signaling pathways and arachidin-1 biosynthesis may be favorable to counteract oxidate stress. Though the highest yields of arachidin-1 and arachidin-3 were found in the hairy root cultures treated with CD+MgCl_2_+H_2_O_2_+MeJA, this treatment also induced secretion of several other stilbenoids which could interfere with the purification steps for arachidin-1 and arachidin-3. However, this four-elicitor treatment could be used to purify these other minor stilbenoids from the extract.

Several studies have shown the preparative separation and isolation of compounds using different parts of the plant. Centrifugal partition chromatography (CPC) and high-speed countercurrent chromatography (HSCCC) techniques were widely used in the preparative separation of plant-specialized metabolites [34,35]. To our knowledge, the semi-preparative isolation of prenylated stilbenoids in large amounts using conventional chromatographic techniques such as column chromatographic techniques and semi-preparative HPLC has not been reported yet. In the present study, we isolated arachidin-1 and arachidin-3 in large quantities from peanut hairy root cultures co-elicited CD+H_2_O_2_ and CD+MeJA using conventional column chromatographic techniques and semi-preparative HPLC. In this study, 258 mg of arachidin-1 and 112 mg of arachidin-3 were isolated from peanut hairy root cultures treated with CD+H_2_O_2_. Similarly, 37 mg of arachidin-1 and 418 mg of arachidin-3 were isolated from peanut hairy root cultures co-treated with CD+MeJA. All compounds were isolated with >95% purity based on the relative area covered by HPLC. To our knowledge, this is the first report of isolation of arachidin-1 and arachidin-3 in such large quantities with high purity from any plant sources.

In this work, we optimized an elicitation procedure to induce the production of the prenylated stilbenoids arachidin-1 and arachidin-3 in peanut hairy root cultures at a larger scale than previously reported. This approach provides a platform to obtain these bioactive compounds in high amounts for further testing in different bioassays in vitro and in vivo.

## 4. Materials and Methods

### 4.1. Hairy Root Cultures of Peanut

Hairy root culture of peanut cv. Hull line 3 was previously established [17] and maintained in 500 mL flasks containing 100 mL MSV medium [17] supplemented with 3% of sucrose at pH 6. All cultures were maintained under continuous darkness on an orbital shaker at 90 rpm at 28 °C. Hairy root tips were subcultured every 15 days into a fresh culture medium.

### 4.2. Growth Kinetics of Peanut Hairy Roots

A total of twelve 2–3 cm long tips were excised from the hairy roots growing in MSV medium and cultured in a 500 mL flask containing 100 mL of MSV liquid medium [17] with 3% sucrose to establish a growth curve. The flask cultures were covered with aluminum foil and maintained on an orbital shaker (Innova 44R, New Brunswick Scientific, Hauppauge, NY, USA) at 90 rpm and 28 °C under continuous darkness. Three flasks were harvested at intervals of 3 days through day 30. The harvested hairy roots were frozen and then lyophilized (Freeze Dry System Freezone 4.5, Labconco™, Kansas City, MO, USA) to obtain the dry weight. The specific growth rate (µ) was calculated as µ = ln (DW_i_/DW_0_)/ Δt where DW_i_ = 18.63 g/L is the average dry weight of the roots in g/L at the end of the exponential growth (day 15), DW_0_ = 0.17 g/L is the average dry weight of inoculum in grams at the start of the exponential growth (day 0), and t is the interval of time (in days) between 0 and i (15 days). Doubling time (T_d_) was calculated as T_d_ = ln (2)/µ. The growth index (GI) was calculated as GI = (DW_i_ − DW_0_)/DW_0_ where DW_i_ is the average dry weight of the roots in g/L at the end of the exponential growth (day 15) and DW_0_ is the average dry weight of inoculum in grams at the start of the exponential growth (day 0). The conductivity and pH were measured from the culture media at each of the time points.

### 4.3. Elicitation of Peanut Hairy Root Cultures

The production of prenylated stilbenoids arachidin-1 and arachidin-3 was evaluated as a function of the different elicitor combinations:

(i) 18 g/L methyl-β-cyclodextrin (CD; CAVASOL^®^ W7 M, Wacker, Munich, Germany);
(ii) 18 g/L CD+3 mM H_2_O_2_ (Thermo Scientific, Waltham, MA, USA);
(iii) 18 g/L CD+125 µM methyl jasmonate (Sigma-Aldrich, St. Louis, MO, USA);
(iv) 18 g/L CD+1 mM MgCl_2_ (Sigma-Aldrich, St. Louis, MO, USA);
(v) 18 g/L CD+125 µM MeJA+3 mM H_2_O_2_+1 mM MgCl_2._

Twelve-day-old peanut hairy root cultures grown in 500 mL flasks containing 100 mL MSV medium were used for elicitation. Prior to elicitation, the spent medium was removed and replaced with 200 mL of fresh MSV medium containing 3% sucrose with different combinations of elicitors as described above. All elicited cultures were incubated on a rotary shaker (90 rpm) at 28 °C under continuous darkness. A control group was also analyzed by only refreshing MSV medium [17] without adding any of the elicitors mentioned above. For the time-course experiment, aliquots of medium from multiple time points (48, 96, 144, and 192 h) were collected from the same flask of elicited culture for HPLC analysis as described below. All treatments were performed in biological triplicates.

### 4.4. Re-Elicitation of Elicited Peanut Hairy Root Cultures

To check the capability of elicited hairy root cultures to produce prenylated stilbenoids arachidin-1 and arachidin-3, elicited hairy root cultures were elicited again with either 18 g/L CD+3 mM H_2_O_2_ or 18 g/L CD+125 µM MeJA. The elicited culture medium was collected aseptically after 192 h of elicitation and elicited again with CD+MeJA or CD+H_2_O_2_ for an additional 192 h. At the end of the re-elicitation period, the culture medium was collected and analyzed by HPLC as described below.

### 4.5. Extraction and Analyses of Arachidin-1 and Arachidin-3

A 900 µL aliquot from the elicited culture medium was extracted with an equal amount of ethyl acetate in a 2 mL microcentrifuge tube by vortexing for 30 s. Then, 500 µL of the upper organic phase was removed, transferred to an amber HPLC vial, and dried under a nitrogen stream using a Reacti-Vap III apparatus (Thermo Fisher Scientific, Waltham, MA, USA). HPLC analyses were performed in an Ultimate 3000 UHPLC system (Thermo Fisher Scientific, Waltham, MA, USA). The extract was resuspended in 500 µL of MeOH and analyzed by reversed-phase HPLC. Briefly, the chromatography was performed in a SunFire^TM^ C18 column (5 µm, 4.6 × 250 mm, UV detection at 340 nm) (Waters, Milford, MA, USA) at 40 °C and a flow rate of 1.0 mL/min. The mobile phase was composed of MeOH (A) and 0.5% HCO_2_H (*v*/*v*) (B). The column was initially calibrated with B for 1 min. Then, a linear gradient was performed using the following program: 60% A to 65% A for 1–20 min, 65% A and 35% B to 100% B for 20–25 min, and 100% B for 25–30 min. Calibration curves for reference compounds were established previously [36]. Similarly, re-elicited culture medium was extracted and analyzed as described above.

### 4.6. Purification of Arachidin-1 from Peanut Hairy Root Cultures Co-Elicited with CD and H_2_O_2_

The extract obtained from peanut hairy root cultures co-elicited with CD+H_2_O_2_ was used to obtain arachidin-1. The hairy root cultures were inoculated into thirty 500 mL flasks containing 100 mL of MSV medium [17]. The cultures were grown till the mid-log stage prior to elicitation. The spent medium was discarded and replaced with 200 mL of MSV medium [17] supplemented with 3% sucrose, 3 mM H_2_O_2_, and 18 g/L CD. The cultures were incubated at 28 °C on a rotary shaker at 90 rpm for 8 days under continuous darkness. After 8 days, the elicited culture media were collected and extracted with ethyl acetate twice at a ratio of 1:1 first time and 2:1 second time. The organic layer was separated from the aqueous layer in a separatory funnel. The organic layer was concentrated in vacuo to afford 2.9 g of extract from 6 L of elicited culture medium. The extract was fractionated by silica gel (Sigma-Aldrich, St. Louis, MO, USA) column chromatography with a step gradient of CHCl_3_–MeOH (1:0, 20:1, 10:1, 4:1, 2:1, 1:1, and 0:1 *v*/*v*). Fraction 3 (10:1) was concentrated to provide a yellowish oil (1.5 g), which was then fractionated by reversed-phase ODS (Nacalai Tesque, Inc., Kyoto, Japan) column chromatography with a gradient of MeCN—0.5% HCO_2_H (2:8, 3:7, 4:6, 5:5, 6:4, 7:3, and 8:2 *v*/*v*). Fraction 5 (6:4) was concentrated and extracted with EtOAc. The organic layer was dried over anhydrous Na_2_SO_4_, filtered, and concentrated to give a semi-pure material (650 mg). Final purification was achieved by semi-preparative HPLC (SunFire C18 OBD™ Prep, Waters, Milford, MA, USA) (10 × 250 mm, 4 mL/min, UV detection at 340 nm) with gradient elution of MeOH/0.5% HCO_2_H solution (50:50 to 90:10, eluting over 30 min) to yield arachidin-1 (258 mg, *t*_R_ 14.3 min), and arachidin-3 (112 mg, *t*_R_ 17.0 min). Arachidin-1 and arachidin-3 were obtained with >95% purity.

### 4.7. Purification of Arachidin-3 from Peanut Hairy Root Cultures Co-Elicited with CD and MeJA

Peanut hairy root culture extract obtained by co-elicitation with CD+MeJA was used to obtain arachidin-3. Cultures were incubated as described above, except that 18 g/L CD and 125 μM MeJA were used as elicitors. After 8 days, the elicited culture media were collected and extracted with ethyl acetate twice at a ratio of 1:1 first time and 2:1 second time. The organic layer was separated from the aqueous layer. The organic layer was concentrated in vacuo to afford 5 g of crude extract from 6 L of elicited culture. The extract was fractionated by silica gel (Sigma-Aldrich, St. Louis, MO, USA) column chromatography with a step gradient of CHCl_3_–MeOH (1:0, 20:1, 10:1, 4:1, 2:1, 1:1, and 0:1 *v*/*v*). Fraction 3 (10:1) was concentrated to provide a yellowish oil (3 g), which was then fractionated by reversed-phase ODS (Nacalai Tesque, Inc., Kyoto, Japan) column chromatography with a gradient of MeCN—0.5% HCO_2_H (2:8, 3:7, 4:6, 5:5, 6:4, 7:3, and 8:2 *v*/*v*). Fraction 5 (6:4) was concentrated and extracted with EtOAc. The organic layer was dried over anhydrous Na_2_SO_4_, filtered, and concentrated to give a semi-pure material (745 mg). Final purification was achieved by semi-preparative HPLC SunFire C18 OBD™ Prep, (Waters, Milford, MA, USA) (10 × 250 mm, 4 mL/min, UV detection at 340 nm) with gradient elution of MeOH/0.5% HCO_2_H solution (50:50 to 90:10, eluting over the course of 30 min) to afford arachidin-1 (37 mg, *t*_R_ 15.1 min), and arachidin-3 (418 mg, *t*_R_ 17.2 min). Arachidin-1 and arachidin-3 were obtained with >95% purity.

### 4.8. Liquid Chromatography–Mass Spectrometry Analysis

The UltiMate 3000 ultra-high-performance liquid chromatography (UHPLC) system (Thermo Scientific, Waltham, MA, USA) was used for chromatography. Chromatographic separation was carried out on a reversed-phase SunFire™ C18 column (5 µm, 4.6 × 250 mm, Waters, Milford, MA, USA) using MeOH (A) and 0.5% HCO_2_H (B) as the mobile phases at a flow rate of 1 mL/min. The linear gradient was performed from 50% A to 90% A for 0–30 min, 90% A to 100% A for 30–35 min, and 100% A for 35–40 min. The injection volume was 10 µl. UV chromatograms were recorded at 340 nm. Mass spectrometry was performed on an LTQ XL linear ion trap (Thermo Scientific, Waltham, MA, USA) with an electrospray ionization (ESI) source. Ultrahigh pure helium (He) was used as the collision gas and high purity nitrogen (N_2_) as the sheath and auxiliary gas. All mass spectra were performed in both positive ion mode with ion spray voltage at 4 kV, sheath gas at 45 arbitrary units (AU), auxiliary gas at 15 AU, and capillary temperature at 300 °C. Full mass scans were recorded in the range *m*/*z* 100–1500. Collision-induced dissociation (CID) was used for the breakage of the molecular ion into smaller fragments. The relative collision energy was set at 35% of the maximum to produce optimum yields of fragment ions. The data were analyzed using the Xcalibur software (Thermo Scientific, Waltham, MA, USA).

## 5. Conclusions

This work demonstrated that a simple elicitation practice using exogenous CD, H_2_O_2_, and MeJA could effectively improve the yields of the biologically active prenylated stilbenoids arachidin-1 and arachidin-3 in peanut hairy root cultures. The results showed that peanut hairy root cultures selectively increase the levels of arachidin-1 or arachidin-3 depending on the elicitation treatment. The combination of CD with MeJA induced higher levels of arachidin-3 whereas CD combined with H_2_O_2_ induced higher levels of arachidin-1. Furthermore, re-elicited peanut hairy root cultures are also liable to produce an elicitor response and are still capable of secreting arachidin-1 and arachidin-3 in substantial amounts into the medium. These results indicate that elicited and re-elicited hairy root cultures are a promising source of extracts for the purification process to obtain a high-purity level of the prenylated stilbenoids arachidin-1 and arachidin-3.

## Figures and Tables

**Table 1:** Prenylated stilbenoids detected in elicited peanut hairy root culture by UHPLC-PDA-ESI-MS^3^.

Arachidins	*t*_R_ (min)	UV Max (nm)	[M+H] ^+^	MS^2^ Ions ^a^	MS^3^ Ions
Arachidin-1	13.84	340	313	**257**	239, 211, 229, 197, 215
Arachidin-3	16.10	335	297	**241**	223, 213, 199, 195

^a^ MS^2^ ions in boldface were the most abundant ions and were subjected to MS^3^ fragmentation.

## Data Availability

The data of this study are available upon request.

## Supplementary Materials

The following supporting information can be downloaded at: https://www.mdpi.com/article/10.3390/molecules27186118/s1, Figure S1: HPLC chromatograms of purified arachidin-1 and arachidin-3 (>95%) from hairy root cultures of peanut elicited with CD+H_2_O_2_; Figure S2: HPLC chromatograms of purified arachidin-1 and arachidin-3 (>95%) from hairy root cultures of peanut elicited with CD+MeJA; Figure S3: LC–MS analysis of arachidin-1 from CD+H_2_O_2_ elicited extract in positive ion mode; Figure S4: LC–MS analysis of arachidin-3 from CD+H_2_O_2_ elicited extract in positive ion mode; Figure S5: LC–MS analysis of arachidin-1 from CD+MeJA elicited extract in positive ion mode; Figure S6: LC–MS analysis of arachidin-3 from CD+MeJA elicited extract in positive ion mode.

## Sample Availability

Samples of arachidin-1 and arachidin-3 are available from the corresponding author.

## References

[1] Sobolev V.S. (2013). Production of phytoalexins in peanut (*Arachis hypogaea*) seed elicited by selected microorganisms. J. Agric. Food Chem..

[2] Yang T., Fang L., Nopo-Olazabal C., Condori J., Nopo-Olazabal L., Balmaceda C., Medina-Bolivar F. (2015). Enhanced production of resveratrol, piceatannol, arachidin-1, and arachidin-3 in hairy root cultures of peanut co-treated with methyl jasmonate and cyclodextrin. J. Agric. Food Chem..

[3] de Bruijn W.J.C., Araya-Cloutier C., Bijlsma J., de Swart A., Sanders M.G., de Waard P., Gruppen H., Vincken J.P. (2018). Antibacterial prenylated stilbenoids from peanut (*Arachis hypogaea*). Phytochem. Lett..

[4] Yang T., Fang L., Sanders S., Jayanthi S., Rajan G., Podicheti R., Thallapuranam S.K., Mockaitis K., Medina-Bolivar F. (2018). Stilbenoid prenyltransferases define key steps in the diversification of peanut phytoalexins. J. Biol. Chem..

[5] Simons R., Gruppen H., Bovee T.F., Verbruggen M.A., Vincken J.-P. (2012). Prenylated isoflavonoids from plants as selective estrogen receptor modulators (phytoSERMs). Food Funct..

[6] de Bruijn W.J.C., Levisson M., Beekwilder J., van Berkel W.J.H., Vincken J.P. (2020). Plant Aromatic Prenyltransferases: Tools for Microbial Cell Factories. Trends Biotechnol..

[7] Valliere M.A., Korman T.P., Woodall N.B., Khitrov G.A., Taylor R.E., Baker D., Bowie J.U. (2019). A cell-free platform for the prenylation of natural products and application to cannabinoid production. Nat. Commun..

[8] Sobolev V.S., Khan S.I., Tabanca N., Wedge D.E., Manly S.P., Cutler S.J., Coy M.R., Becnel J.J., Neff S.A., Gloer J.B. (2011). Biological activity of peanut (*Arachis hypogaea*) phytoalexins and selected natural and synthetic stilbenoids. J. Agric. Food Chem..

[9] Abbott J.A., Medina-Bolivar F., Martin E.M., Engelberth A.S., Villagarcia H., Clausen E.C., Carrier D.J. (2010). Purification of resveratrol, arachidin-1, and arachidin-3 from hairy root cultures of peanut (*Arachis hypogaea*) and determination of their antioxidant activity and cytotoxicity. Biotechnol. Prog..

[10] Chen L.-G., Zhang Y.-Q., Wu Z.-Z., Hsieh C.-W., Chu C.-S., Wung B.-S. (2017). Peanut arachidin-1 enhances Nrf2-mediated protective mechanisms against TNF-α-induced ICAM-1 expression and NF-κB activation in endothelial cells. Int. J. Mol. Med..

[11] Ball J.M., Medina-Bolivar F., Defrates K., Hambleton E., Hurlburt M.E., Fang L., Yang T., Nopo-Olazabal L., Atwill R.L., Ghai P. (2015). Investigation of stilbenoids as potential therapeutic agents for rotavirus gastroenteritis. Adv. Virol..

[12] Brents L.K., Medina-Bolivar F., Seely K.A., Nair V., Bratton S.M., Nopo-Olazabal L., Patel R.Y., Liu H.N., Doerksen R.J., Prather P.L. (2012). Natural prenylated resveratrol analogs arachidin-1 and-3 demonstrate improved glucuronidation profiles and have affinity for cannabinoid receptors. Xenobiotica.

[13] Chang J.C., Lai Y.H., Djoko B., Wu P.L., Liu C.D., Liu Y.W., Chiou R.Y. (2006). Biosynthesis enhancement and antioxidant and anti-inflammatory activities of peanut (*Arachis hypogaea* L.) arachidin-1, arachidin-3, and isopentadienylresveratrol. J. Agric. Food Chem..

[14] Liu Z., Wu J., Huang D. (2013). New stilbenoids isolated from fungus-challenged black skin peanut seeds and their adipogenesis inhibitory activity in 3T3-L1 cells. J. Agric. Food Chem..

[15] Mohammadhosseinpour S., Ho L.-C., Fang L., Xu J., Medina-Bolivar F. (2022). Arachidin-1, a Prenylated Stilbenoid from Peanut, Induces Apoptosis in Triple-Negative Breast Cancer Cells. Int. J. Mol. Sci..

[16] Medina-Bolivar F., Condori J., Rimando A.M., Hubstenberger J., Shelton K., O’Keefe S.F., Bennett S., Dolan M.C. (2007). Production and secretion of resveratrol in hairy root cultures of peanut. Phytochemistry.

[17] Condori J., Sivakumar G., Hubstenberger J., Dolan M.C., Sobolev V.S., Medina-Bolivar F. (2010). Induced biosynthesis of resveratrol and the prenylated stilbenoids arachidin-1 and arachidin-3 in hairy root cultures of peanut: Effects of culture medium and growth stage. Plant Physiol. Biochem..

[18] Fang L., Yang T., Medina-Bolivar F. (2020). Production of prenylated stilbenoids in hairy root cultures of peanut (*Arachis hypogaea*) and its wild relatives *A. ipaensis* and *A. duranensis* via an optimized elicitation procedure. Molecules.

[19] Sobolev V.S., Cole R.J., Dorner J.W., Yagen B. (1995). Isolation, purification, and liquid chromatographic determination of stilbene phytoalexins in peanuts. J. AOAC Int..

[20] Sobolev V.S., Neff S.A., Gloer J.B. (2010). New dimeric stilbenoids from fungal-challenged peanut (*Arachis hypogaea*) seeds. J. Agric. Food Chem..

[21] Yang T., Fang L., Rimando A.M., Sobolev V., Mockaitis K., Medina-Bolivar F. (2016). A stilbenoid-specific prenyltransferase utilizes dimethylallyl pyrophosphate from the plastidic terpenoid pathway. Plant Physiol..

[22] Gutierrez-Valdes N., Häkkinen S.T., Lemasson C., Guillet M., Oksman-Caldentey K.M., Ritala A., Cardon F. (2020). Hairy root cultures—A versatile tool with multiple applications. Front. Plant Sci..

[23] Sobolev V.S. (2008). Localized production of phytoalexins by peanut (*Arachis hypogaea*) kernels in response to invasion by *Aspergillus* species. J. Agric. Food Chem..

[24] Sobolev V.S., Neff S.A., Gloer J.B. (2009). New stilbenoids from peanut (*Arachis hypogaea*) seeds challenged by an *Aspergillus caelatus* strain. J. Agric. Food Chem..

[25] Aisyah S., Gruppen H., Slager M., Helmink B., Vincken J.-P. (2015). Modification of prenylated stilbenoids in peanut (*Arachis hypogaea*) seedlings by the same fungi that elicited them: The fungus strikes back. J. Agric. Food Chem..

[26] Cardillo A.B., Perassolo M., Giulietti A.M., Rodriguez-Talou J. (2021). Cyclodextrins: A tool in plant cell and organ culture bioprocesses for the production of secondary metabolites. Plant Cell Tissue Organ Cult..

[27] Marsh Z., Yang T., Nopo-Olazabal L., Wu S., Ingle T., Joshee N., Medina-Bolivar F. (2014). Effect of light, methyl jasmonate and cyclodextrin on production of phenolic compounds in hairy root cultures of *Scutellaria lateriflora*. Phytochemistry.

[28] Ho T.-T., Murthy H.N., Park S.-Y. (2020). Methyl jasmonate induced oxidative stress and accumulation of secondary metabolites in plant cell and organ cultures. Int. J. Mol. Sci..

[29] Gill S.S., Tuteja N. (2010). Reactive oxygen species and antioxidant machinery in abiotic stress tolerance in crop plants. Plant Physiol. Biochem..

[30] Moomaw A.S., Maguire M.E. (2008). The Unique Nature of Mg^2+^ Channels. Physiology.

[31] Sabater-Jara A.B., Onrubia M., Moyano E., Bonfill M., Palazón J., Pedreño M.A., Cusidó R.M. (2014). Synergistic effect of cyclodextrins and methyl jasmonate on taxane production in *Taxus* x media cell cultures. Plant Biotechnol. J..

[32] Almagro L., Gutierrez J., Pedreño M.A., Sottomayor M. (2014). Synergistic and additive influence of cyclodextrins and methyl jasmonate on the expression of the terpenoid indole alkaloid pathway genes and metabolites in *Catharanthus roseus* cell cultures. Plant Cell Tissue Organ Cult..

[33] Nopo-Olazabal C., Condori J., Nopo-Olazabal L., Medina-Bolivar F. (2014). Differential induction of antioxidant stilbenoids in hairy roots of *Vitis rotundifolia* treated with methyl jasmonate and hydrogen peroxide. Plant Physiol. Biochem..

[34] Lima Á.S., de Oliveira B.S., Shabudin S.V., Almeida M., Freire M.G., Bica K. (2021). Purification of anthocyanins from grape pomace by centrifugal partition chromatography. J. Mol. Liq..

[35] Zhao W., Wang Y., Hao W., Yang H., Song X., Zhao M., Peng S.J. (2015). Preparative isolation and purification of urolithins from the intestinal metabolites of pomegranate ellagitannins by high-speed counter-current chromatography. J. Chromatogr. B Anal. Technol. Biomed. Life Sci..

[36] Gajurel G., Hasan R., Medina-Bolivar F. (2021). Antioxidant assessment of prenylated stilbenoid-rich extracts from elicited hairy root cultures of three cultivars of peanut (*Arachis hypogaea*). Molecules.

