# Supplementary material for: Induction of the Prenylated Stilbenoids Arachidin-1 and Arachidin-3 and Their Semi-Preparative Separation and Purification from Hairy Root Cultures of Peanut (Arachis hypogaea L.)
Source: Molecules. 2022 Sep 19;27(18):6118. doi: 10.3390/molecules27186118 (PMC9504991; doi:10.3390/molecules27186118)
Supplement: Supplementary file 1 [file molecules-27-06118-s001.zip › molecules-1899859-supplementary.pdf]

Supplementary Materials

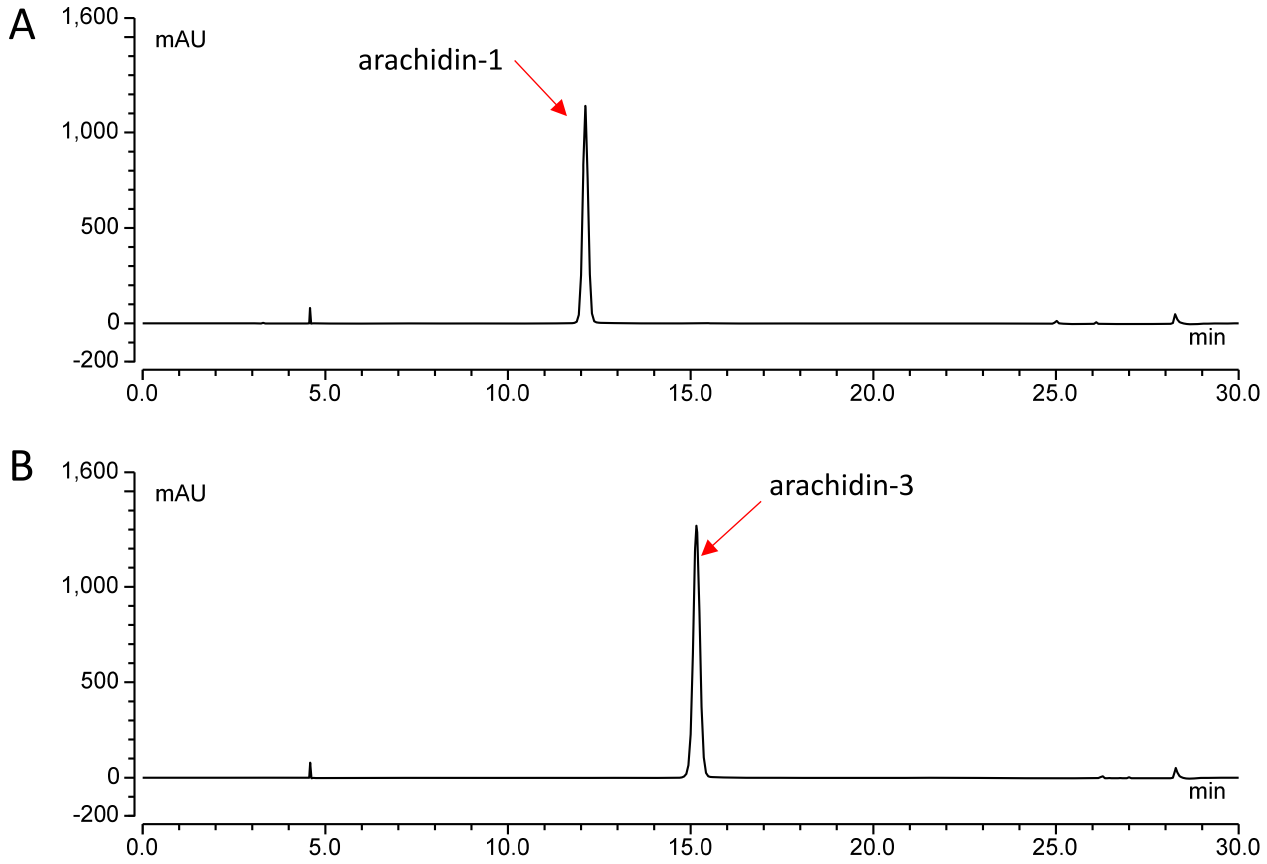

Figure S1. HPLC chromatograms of purified (**A**) arachidin-1 and (**B**) arachidin-3 (>95%) from hairy root cultures of peanut elicited with CD+H_2O_2. Detection was done at 340 nm.

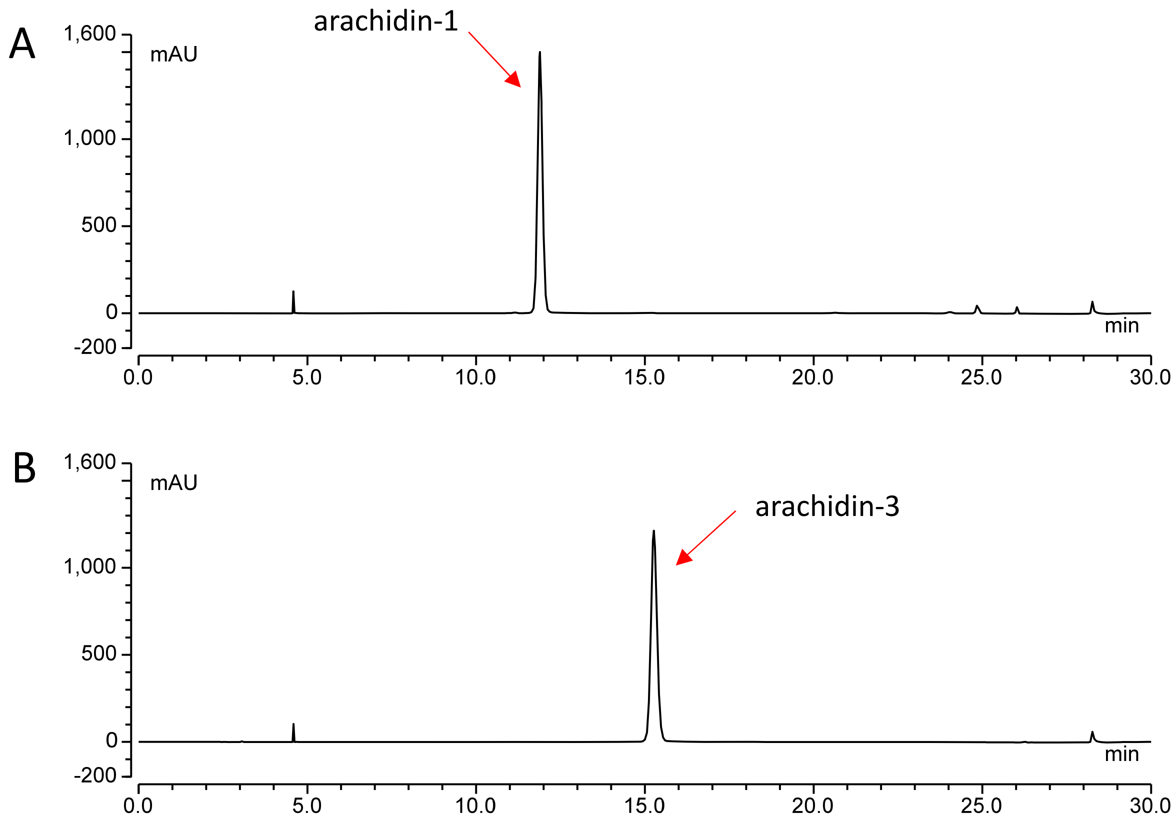

Figure S2. HPLC chromatograms of purified (**A**) arachidin-1 and (**B**) arachidin-3 (>95%) from hairy root cultures of peanut elicited with CD+MeJA. Detection was done at 340 nm.

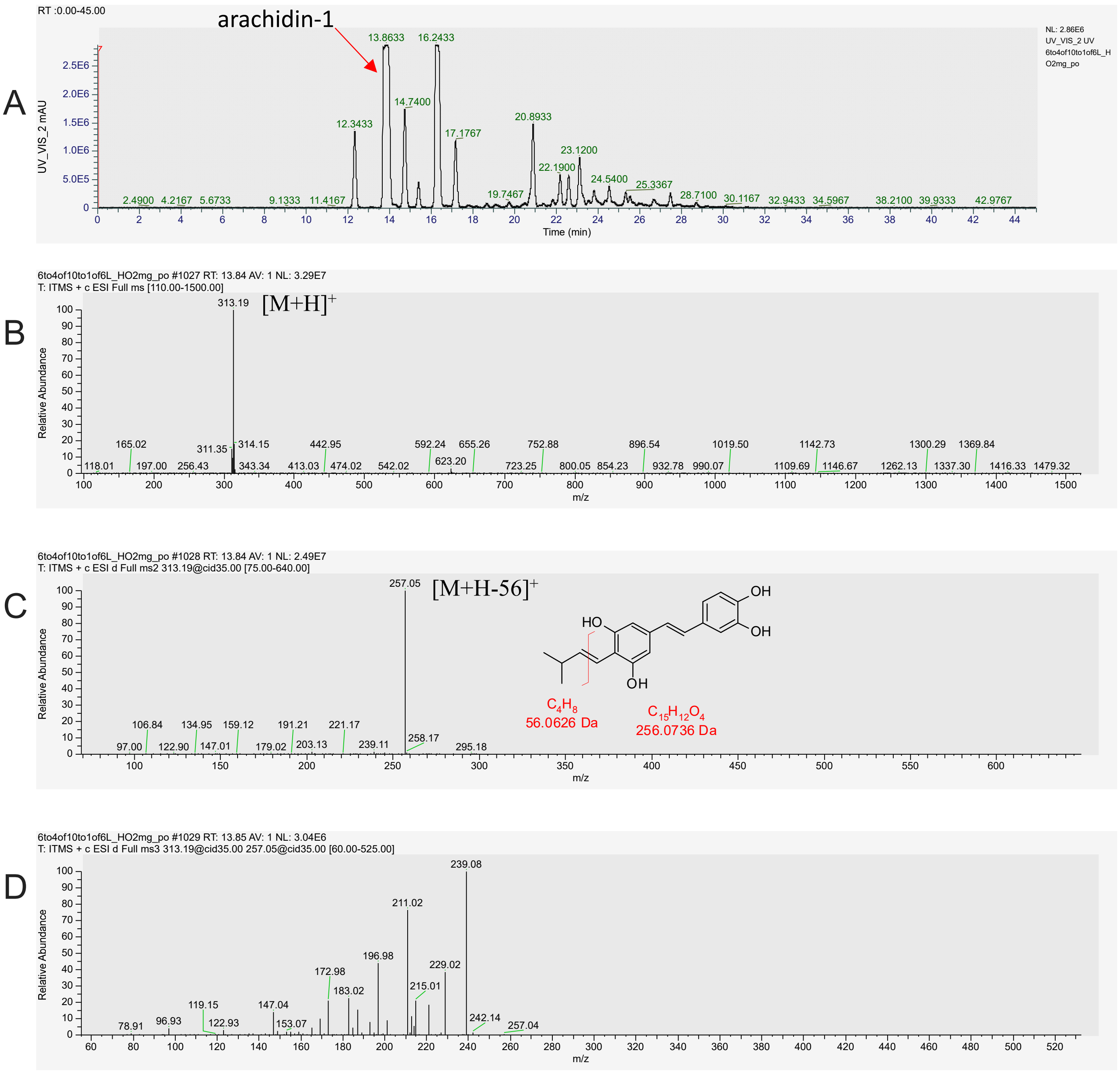

Figure S3. LC-MS analysis of arachidin-1 from CD+H_2O_2 elicited extract in positive ion mode. (**A**) HPLC chromatogram; (**B**) MS ion chromatogram; (**C**) MS^2 ion chromatogram; (**D**) MS^3 ion chromatogram.

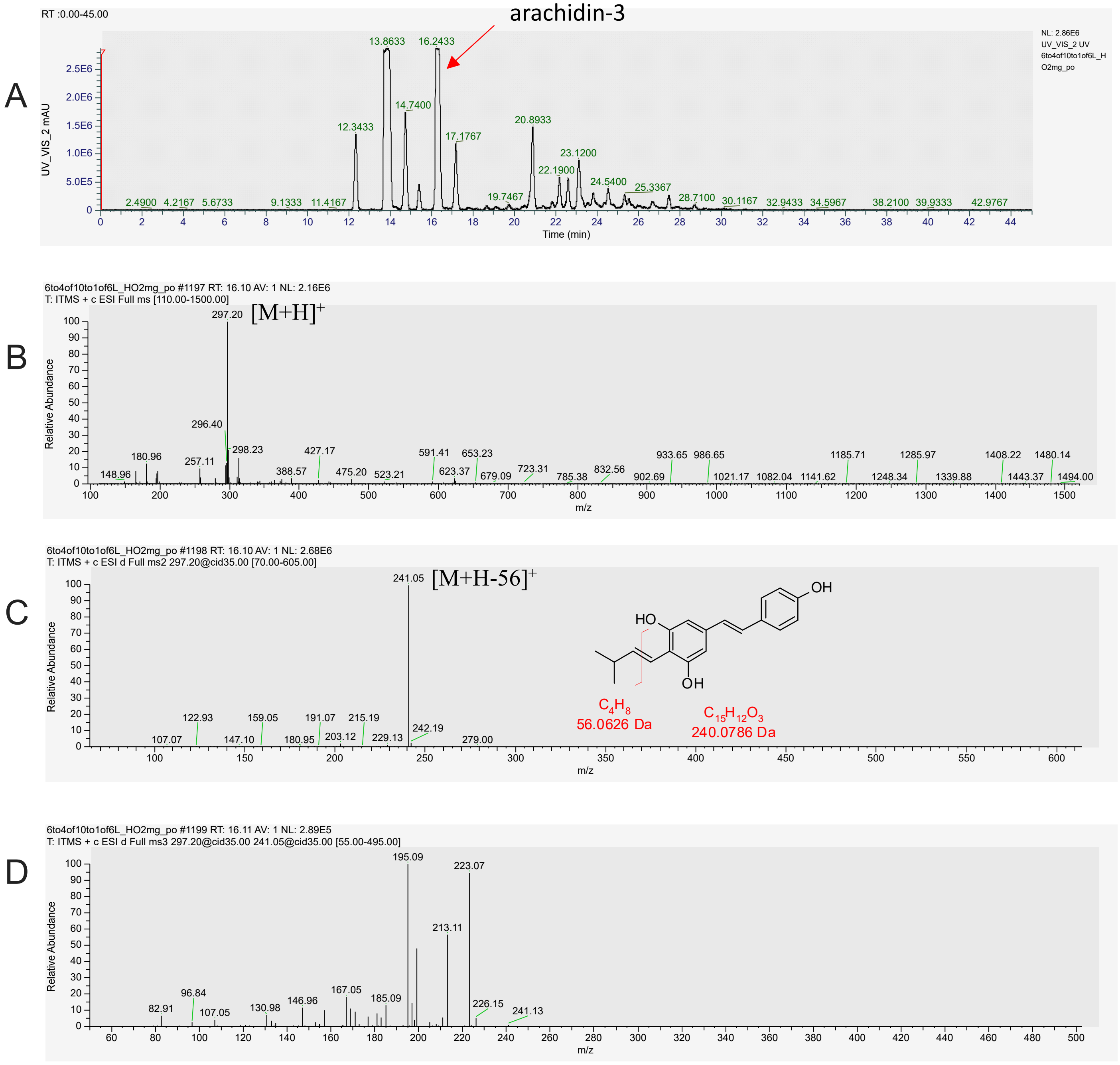

Figure S4. LC-MS analysis of arachidin-3 from CD+H_2O_2 elicited extract in positive ion mode. (**A**) HPLC chromatogram; (**B**) MS ion chromatogram; (**C**) MS^2 ion chromatogram; (**D**) MS^3 ion chromatogram.

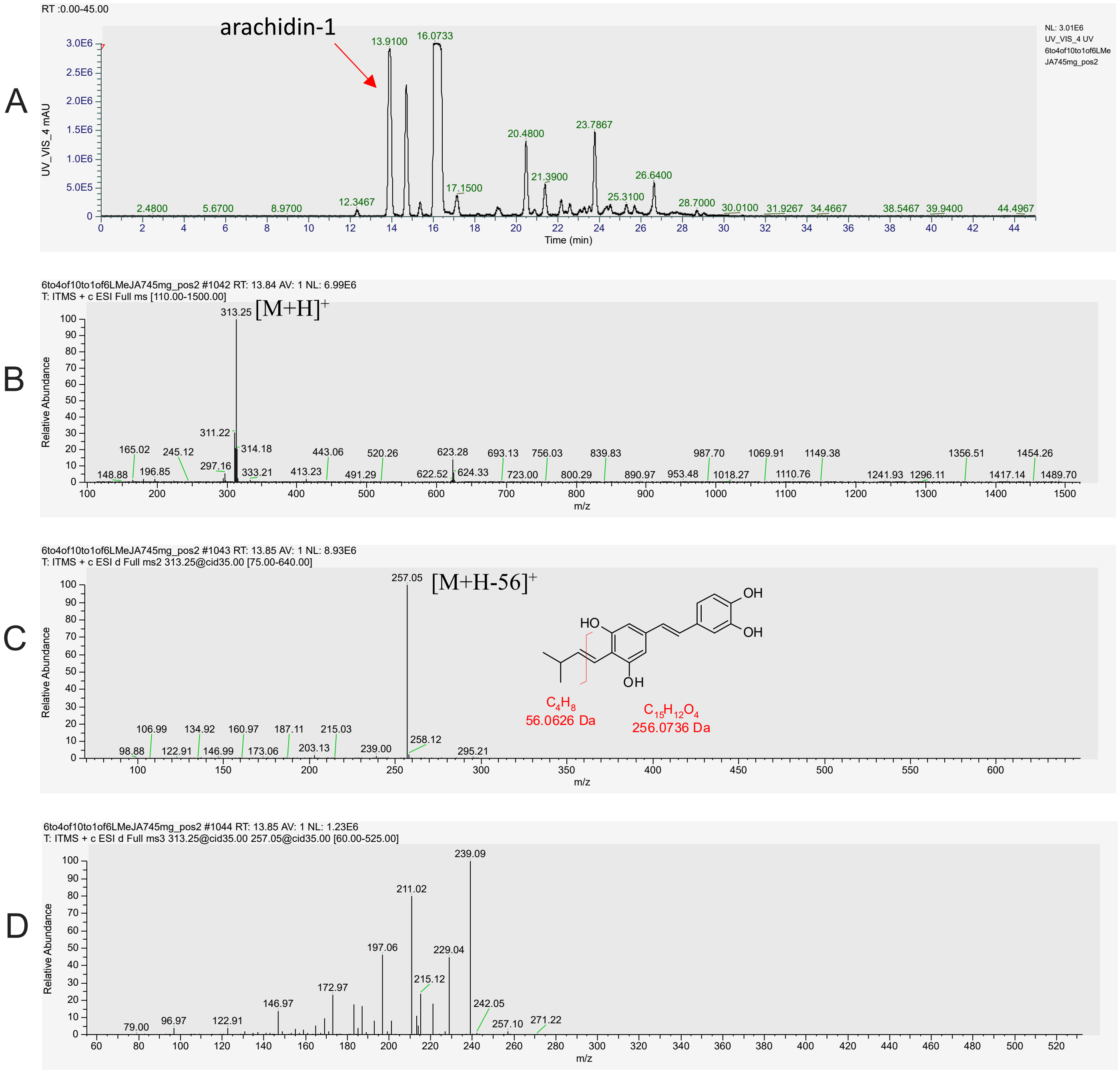

Figure S5. LC-MS analysis of arachidin-1 from CD+MeJA elicited extract in positive ion mode. (**A**) HPLC chromatogram; (**B**) MS ion chromatogram; (**C**) MS^2 ion chromatogram; (**D**) MS^3 ion chromatogram.

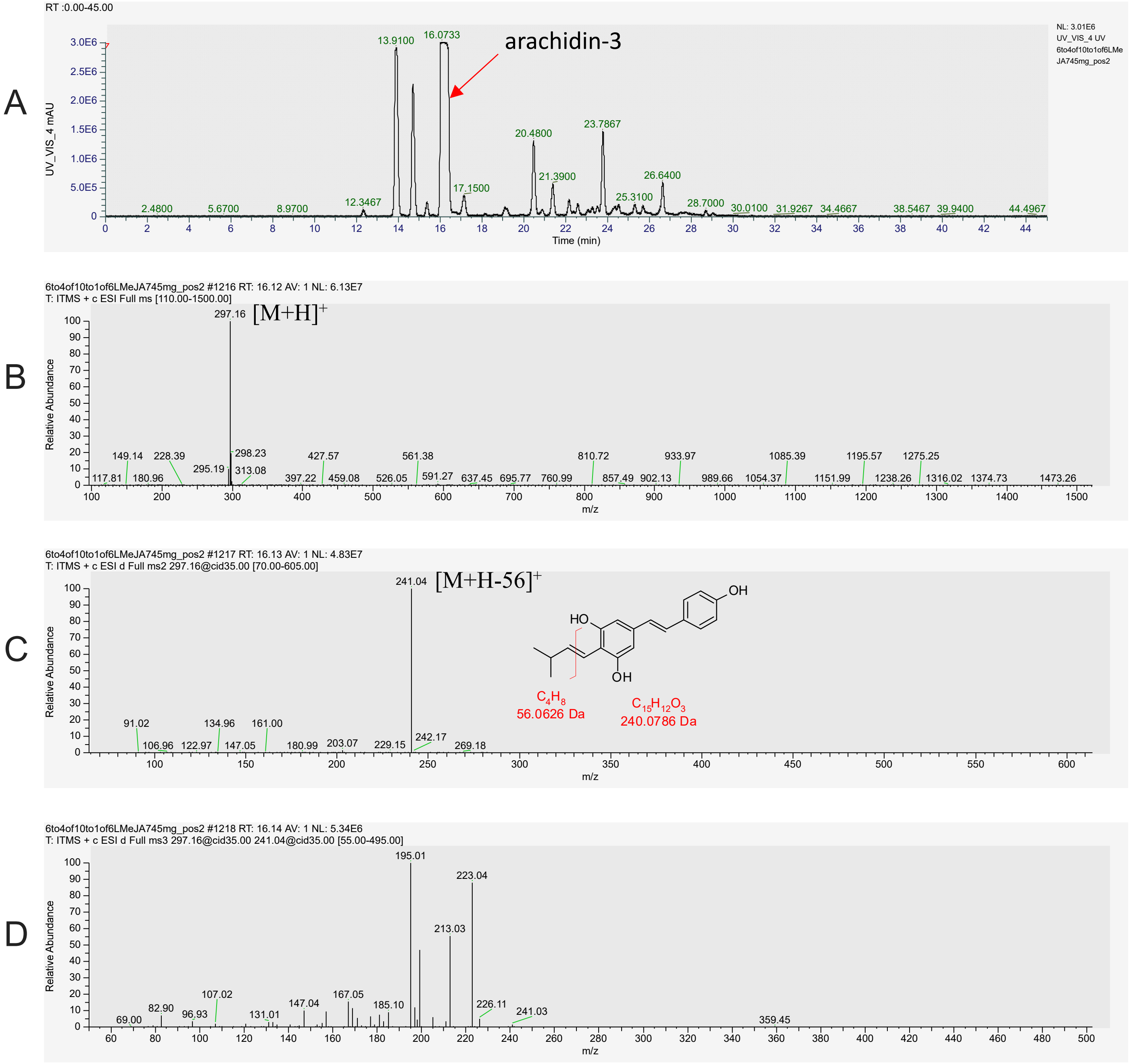

Figure S6. LC-MS analysis of arachidin-3 from CD+MeJA elicited extract in positive ion mode. (**A**) HPLC chromatogram (**B**) MS ion chromatogram; (**C**) MS^2 ion chromatogram; (**D**) MS^3 ion chromatogram.
